# Anti-Inflammatory Thioredoxin Family Proteins for Medicare, Healthcare and Aging Care

**DOI:** 10.3390/nu9101081

**Published:** 2017-09-29

**Authors:** Junji Yodoi, Yoshiyuki Matsuo, Hai Tian, Hiroshi Masutani, Takashi Inamoto

**Affiliations:** 1 Japan Biostress Research Promotion Alliance (JBPA), 1-6 Shogoin Kawahara-cho, Sakyo-ku, Kyoto 606-8397, Japan; tianhai2820044@yahoo.co.jp (H.T.); inamoto@tenriyorozu-u.ac.jp (T.I.); 2 Institute for Virus Research, Kyoto University, 53 Shogoin Kawahara-cho, Sakyo-ku, Kyoto 606-8507, Japan; 3 Department of Human Stress Response Science, Institute of Biomedical Science, Kansai Medical University, 2-5-1 Shin-machi, Hirakata, Osaka 573-1010, Japan; matsuoys@hirakata.kmu.ac.jp; 4 Department of Anatomy, Basic Medicine Science, Medical College, Shaoxing University, No 900 Cengnan Avenue, Shaoxing 312000, China; 5 Terni Health Care University, 80-1 Bessho-cho, Tenri, Nara 632-0018, Japan; h.masutani@tenriyorozu-u.ac.jp

**Keywords:** inflammation, oxidative stress, redox, thioredoxin, thioredoxin-interacting protein

## Abstract

Human thioredoxin (TRX) is a 12-kDa protein with redox-active dithiol in the active site -Cys-Gly-Pro-Cys-, which is induced by biological stress due to oxidative damage, metabolic dysfunction, chemicals, infection/inflammation, irradiation, or hypoxia/ischemia-reperfusion. Our research has demonstrated that exogenous TRX is effective in a wide variety of inflammatory diseases, including viral pneumonia, acute lung injury, gastric injury, and dermatitis, as well as in the prevention and amelioration of food allergies. Preclinical and clinical studies using recombinant TRX (rhTRX) are now underway. We have also identified substances that induce the expression of TRX in the body, in vegetables and other plant ingredients. Skincare products are being developed that take advantage of the anti-inflammatory and anti-allergic action of TRX. Furthermore, we are currently engaged in the highly efficient production of pure rhTRX in several plants, such as lettuce, grain and rice.

## 1. Introduction

Thioredoxin (TRX) is a small protein with a catalytically active dithiol site (Cys-Gly-Pro-Cys) found in a variety of life forms on earth, including bacteria, plants, and animals [1,2,3]. The active site contains two cysteines (dithiol) undergoing reversible redox change between an oxidized disulfides (-S-S-) and a reduced dithiol (-SH, -SH). TRX in the reduced state catalyzes the cleavage of disulfide bonds in the target proteins, and it becomes oxidized after completion of the reaction. Oxidized TRX is restored to the reduced form by the nicotinamide adenine dinucleotide phosphate (NADPH)-dependent thioredoxin reductase. TRX quenches reactive oxygen species by coupling with TRX-dependent peroxidases, or peroxiredoxins [4]. 

**Figure 1:**
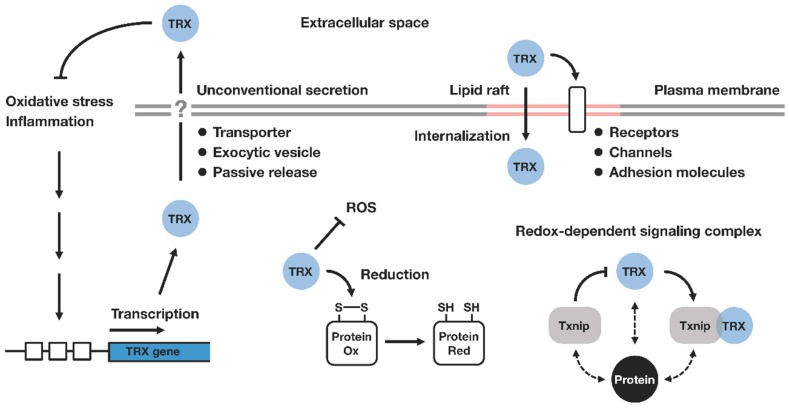
Thioredoxin-mediated redox regulation. Thioredoxin (TRX) is transcriptionally upregulated in response to environmental or pathological factors associated with oxidative stress. TRX catalyzes the cleavage of protein disulfide bonds, thereby contributing to the maintenance of cellular redox homeostasis. TRX is secreted into the extracellular compartment via ER/Golgi-independent mechanisms (unconventional or nonclassical secretion), where it exhibits its protective effects against inflammation. Extracellular TRX is associated with membrane lipid rafts, where it can control the redox state of cell surface molecules, and influence the downstream signaling pathway. The internalization of extracellular TRX is also mediated through lipid rafts. Intracellularly, TRX interacts with a number of signaling molecules in a redox-dependent fashion. Txnip (also known as TBP-2 or VDUP1) has been identified as a negative regulator of TRX and the binding of Txnip inhibits the reducing activity of TRX. TRX and Txnip form a redox-sensitive signaling complex termed ‘redoxisome’, which may play a central role in the regulation of diverse biological processes ranging from metabolic and immunological pathways to inflammatory response and tumorigenesis. Ox, oxidized; Red, reduced; ROS, reactive oxygen species; TRX, thioredoxin; Txnip, thioredoxin interacting protein.

Subsequent studies have shown that TRX is involved in various redox-dependent cellular processes, such as gene expression, signal transduction, cell growth and apoptosis. Various kinds of TRX targets and interacting molecules have been reported. TRX exerts a variety of regulatory actions maintaining the cellular, as well as extracellular, redox homeostasis (Figure 1).

## 2. Background of TRX Research Associated with Human Diseases

In the early 1960s, TRX was reported as a ribonucleotide-reducing co-enzyme by the Karolinska Institute [5]. In addition, Immunoglobulin E (IgE) was discovered by Kimishige Ishizaka’s group in Denver Colorado, USA as the “regain” antibody [6], which was confirmed to be identical to the unknown myeloma protein studied by Bennich and Johansson’s group in Sweden [7]. Human TRX was first identified as a secretory autocrine and IL-2 receptor inducing protein, adult T cell leukemia (ATL)-derived factor (ADF) from the culture supernatant of human T-lymphotropic virus type I (HTLV-I)-transformed T cell lines [8]. ATL characterized by abnormal leukemia cells having multi-convoluted nuclei and T cell properties was reported in Japan in the early 1970s. ATL cases were frequent in the southern regions of Japan, and soon proved to be associated with retrovirus HTLV-I [9,10].

The human TRX gene is located on chromosome 9 (9q31), and the coding sequence of TRX consists of five exons. The promoter region contains several regulatory elements, such as antioxidant responsive element (ARE), which enable the gene to be responsive to various external stimuli [11]. The TRX gene encodes the protein of 105 amino acids, and TRX resides mainly in a cytosolic compartment. Although TRX lacks an N-terminal signal peptide for the vesicular secretion pathway, it is released by cells under various stress conditions, where it exhibits cytokine and chemokine-like activities [12]. It seems that the extracellular function of TRX is not mediated through the canonical ligand–receptor interaction, and no specific cell surface receptors for TRX have been identified. It has been shown that TRX regulates the redox status of target cell surface molecules, such as CD4, CD30, and a type of transient potential channel, thereby controlling the downstream signaling [13,14,15]. Extracellular TRX may also be taken up by cells through membrane lipid rafts, and exert its effect intracellularly [16].

TRX is exported to the extracellular space by an unconventional ER/Golgi-independent pathway, which has been poorly characterized. No interaction has been found between TRX and any membranous elements or vesicles [17,18]. There was a report suggesting the partial involvement of inflammatory caspase-1 in the release of TRX [19], but the precise mechanism that mediates the secretion of TRX still remains unknown.

Increased levels of extracellular TRX have been reported in many pathological conditions associated with oxidative stress (Table 1) [20,21,22,23,24,25,26,27,28,29,30,31,32,33,34,35,36,37,38,39,40,41,42,43,44,45,46,47,48,49,50,51]. The elevation of TRX in plasma or serum suggests the potential utility of TRX as a useful clinical parameter for inflammatory disorders. It has also been reported that TRX is highly expressed in salivary glands of Sjögren’s syndrome patients [48]. TRX was secreted into saliva, and the levels of salivary TRX were correlated well with the severity of the disease, implying that TRX could be used as a noninvasive marker to reflect the oxidative tissue damage.

**Figure 2:**
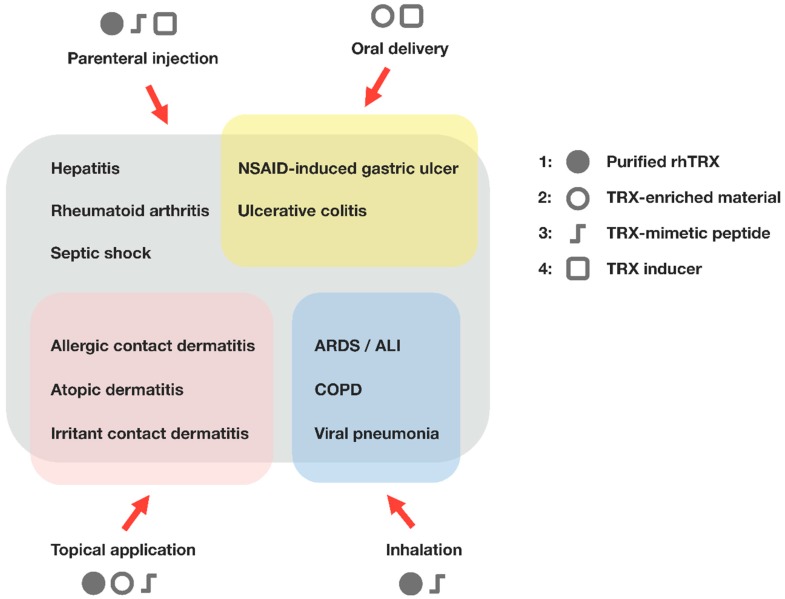
Therapeutic applications of thioredoxin. Administration of TRX suppresses the excessive inflammatory response and any associated tissue injury, indicating the benefits of TRX for the treatment of inflammatory conditions. Four different types of thioredoxin-based therapeutics and the possible routes of administration are shown. (1) Recombinant human TRX (rhTRX). Expression systems for producing rhTRX have been established in *E. coli* and transgenic plants; (2) TRX-enriched material. Yeast cells secrete TRX in response to ethanol treatment, and the yeast-derived protein extracts with high TRX content retain anti-inflammatory activity; (3) TRX-mimetic peptide [60]. Smaller peptide mimetics may have a potential advantage over rhTRX, with respect to production cost and delivery efficiency; (4) TRX inducer. TRX-inducing compounds that increase endogenous TRX levels may also offer protection against inflammation and oxidative stress. ALI, acute lung injury; ARDS, acute respiratory distress syndrome; COPD, chronic obstructive pulmonary disease; NSAID, nonsteroidal anti-inflammatory drug.

Transgenic overexpression of TRX and the systemic administration of recombinant human thioredoxin (rhTRX) are effective in a wide variety of in vivo inflammatory disease models, such as viral pneumonia, acute lung injury, pancreatitis, myocarditis, chronic obstructive pulmonary disease, and indomethacin-induced gastric injury (Figure 2). Elevated TRX levels in several primary tumors may suggest that TRX up-regulation in the cytosol can overwhelm the benefits of antioxidant/anti-inflammatory properties in a particular pathological condition, where compounds targeting TRX and TRX reductase can be effective [52,53,54,55]. However, a number of preclinical studies have demonstrated that extracellular TRX has a cytoprotective function under oxidative and inflammatory conditions, and no apparent evidence of adverse effects or undesirable symptoms have yet been found. The protective effects of TRX against inflammation and its potential clinical utility have been extensively reviewed elsewhere [56,57,58,59]. In this review, we describe the recent findings regarding the therapeutic applications of TRX and the strategies for TRX supplementation. In addition to the clinical use of TRX, we also discuss its potential utility as an attractive functional component of cosmetic products and dietary supplements.

## 3. Mucosa and Skin Inflammation

In TRX-transgenic mice, allergic contact dermatitis (ACD), irritant contact dermatitis (ICD) and ultraviolet light-induced dermatitis were unequivocally suppressed [61,62]. Transgenic overexpression of TRX in mice suppressed the allergic reaction and inflammation in an experimental ACD model. The migratory function of cutaneous dendritic cells and the subsequent antigen-specific proliferation of lymph node cells after dinitrofluorobenzene (DNFB) sensitization were equivalent in both TRX-transgenic mice and wild type mice [61]. Thus, the overproduction of TRX in mice did not affect the primary immune response in the induction phase of ACD, whereas skin inflammation was suppressed by diminishing the infiltration of neutrophils in TRX-transgenic mice after elicitation challenge with DNFB. There were no apparent differences in immune cell populations between TRX-transgenic and wild type animals [63]. These findings indicate that TRX exerts an anti-inflammatory effect in the elicitation phase of ACD, suggesting that the anti-inflammatory mechanism of TRX is different from the mechanisms associated with other anti-inflammatory agents, such as the glucocorticoids, which regulate the inflammatory reaction in association with the suppression of immune responses.

The protective effects of exogenously applied TRX have also been demonstrated in an irritant contact dermatitis (ICD) model. The ICD mouse model induced by croton oil has been widely accepted as a useful pharmacological model for the investigation of new anti-inflammatory drugs [64]. Croton oil contains phorbol 12-myristate 13-acetate (PMA) and other phorbol esters as main irritant agents. Croton oil is known to cause significant inflammatory responses by inducing the release of inflammatory cytokines produced by keratinocytes, as characterized by edema, neutrophil infiltration, prostaglandins production, and increases in vascular permeability. Topically applied rhTRX inhibited the production and release of pro-inflammatory mediators at the site of the inflammation, thereby suppressing ICD [65]. The local application of TRX proteins may be a promising therapeutic strategy to prevent a variety of skin and mucosal inflammatory disorders. Based on these findings, skincare products are being developed that take advantage of the anti-allergic and anti-aging action of TRX.

## 4. Oral Delivery of TRX

Transcription factors containing sulfhydryl groups, such as the activator protein 1 (AP-1) and nuclear factor-κB (NF-κB), increase DNA-binding activity through the change in the redox state of the cysteine residues by TRX directly or indirectly [66,67]. Apoptosis signal-regulating kinase-1 (ASK1), a MAP kinase kinase kinase, is inhibited by being bound to reduced TRX [68]. These functions suggested that TRX plays a defensive role against several diseases, including gastrointestinal disease. Previous studies have shown attenuation of dextran sulfate sodium (DSS)-induced colitis [37], *Helicobacter felis*-induced gastritis [69] and indomethacin-induced gastric mucosal injury [70] in TRX-overexpressing transgenic mice or mice after systemic administration of rhTRX. Recently, Nakajima et al. reported that oral administration of sake yeast extracts with a high TRX content reduced indomethacin-induced gastric injury [71], suggesting that orally administered TRX, and not merely endogenous TRX or injected rhTRX, can protect the gastric mucosa. However, no studies have been conducted to investigate how long orally administered TRX remains in the stomach. Taketani et al. demonstrated that orally administered TRX derived from yeast, which is commonly used in fermented foods, has a protective effect on the gastric mucosa both in in vitro and in vivo models (water-immersion restraint stress and HCl/ethanol-induced gastric ulcer models) [72]. DNA microarray analysis revealed the upregulation of genes related to tissue repair in ulcer regions of rats administered with yeast TRX. These results demonstrated that oral administration could be an alternative option for targeted delivery of TRX to the sites of inflammation. We are now engaged in the highly efficient production of TRX in several plants, such as lettuce [73], grain and rice, and they should provide a feasible source for oral delivery of TRX.

## 5. TRX-Inducing Principles

Given its nature to respond to oxidative stresses, TRX expression can be induced by a variety of physiochemical stimuli, including virus infection, mitogen, UV-irradiation, hydrogen peroxide and ischemia-reperfusion, which we have extensively reviewed [58,74,75]. Natural metabolic or endocrine substances including hemin, estrogen, prostaglandins, sulforaphane, and cAMP can also induce the expression and secretion of TRX [76,77,78]. Geranylgeranylacetone (GGA), an acyclic polyisoprenoid used as an anti-ulcer drug, and tert-butylhydroquinone (tBHQ), an electrophile stressor, can also induce TRX expression [79,80,81]. A series of stress-responsive elements in the promoter region have been identified, including the oxidative stress response element (ORE), antioxidant responsive element (ARE), cAMP responsive element (CRE), xenobiotics responsive element (XRE) and Sp1 [76,77,81,82,83]. Recently, we showed that fragrant unsaturated aldehydes from edible plants are novel TRX inducers, through the activation of the ARE element in the promoter region, and that they may be beneficial for protection against oxidative stress-induced cellular damage [84].

## 6. Thioredoxin Interacting Protein (Txnip/TBP-2/VDUP1)

Thioredoxin interacting protein (Txnip) was originally cloned as a vitamin D3 target gene (named the vitamin D3 upregulated protein, VDUP1). This molecule has emerged as a key component of the cellular redox-regulation, since it was identified as a binding partner of TRX and further, suggested as an endogenous TRX inhibitor (named the thioredoxin binding protein-2, TBP-2) [85]. Two Txnip cysteines are important for thioredoxin binding through a disulfide exchange reaction between oxidized Txnip and reduced TRX [86]. This clear evidence suggests that the TRX–Txnip complex is important for various redox-dependent cell functions. Interestingly, Txnip is a member of the α-arrestin protein family (ARRDC1-5 and Txnip) containing two characteristic arrestin-like domains with the PxxP sequence, which is a binding motif for SH3-domain containing proteins, and the PPxY sequence, which is known as the binding motif for the WW-domain [86,87,88,89,90]. Since Txnip has specific arrestin-like domains that are responsible for protein–protein binding, a number of studies have identified various interacting partners for Txnip, such as the importin-α, SMRT-mSin3-HDAC corepressor, JAB1, E3 ubiquitin ligase itch, Mybbp1a and NLRP3, as well as TRX [91,92,93,94,95]. These findings raise the possibility that Txnip may play a scaffolding role in the signal complex. Txnip is highlighted in the metabolic regulation, since the molecule was identified as a nonsense mutation gene in Hcb-19 mice, which is known as the familial combined hyperlipidemic model [96]. Hcb-19 mice have decreased CO_2_ production, but increased ketone body synthesis, and the evidence highlighted the fact that altered redox status by TRX/Txnip down-regulates the lipid metabolism, such as the citric-acid cycle, sparing fatty acids for triglyceride and ketone body production [97].

Previously, we reported that gene targeting disruption of Txnip (Txnip KO) in mice resulted in a predisposition to death with severe bleeding, hypoglycemia, hyperinsulinemia and liver steatosis during fasting [98]. Txnip gene expression is induced in fasting, and the key transcription regulator peroxisome proliferator activated receptor-α (PPAR-α) and sterol response element-binding protein (SREBP) signaling are dysregulated in the liver of Txnip KO during feeding–fasting nutritional transition [99]. Txnip expression is widely regulated by nutritional status, obesity, high glucose, amino acids, nuclear receptor signal and AMPK [87,94,99,100,101,102,103,104,105]. These results clearly suggest that Txnip is an important molecule to regulate glucose and lipid homeostasis. 

## 7. TRX/Txnip; Redoxisome, a Redox-Related Signal Complex

The TRXs system plays an important role in maintaining a reducing environment in cells. We first identified Txnip/TBP-2/VDUP1 as an endogenous TRX binding and inhibiting protein [85]. Interestingly, Txnip bound to reduced TRX, but not to oxidized TRX, nor to mutant TRX, in which two redox-active cysteine residues were substituted by serine [85]. Since the disulfide exchange reaction between oxidized Txnip and reduced TRX (Txnip and TRX form a stable disulfide-linked complex) is known as the essential event for the interaction between Txnip and TRX [86], these two Txnip cysteines are important for TRX binding. These cysteines are not conserved in the broader family of arrestin domain-containing proteins, suggesting that the TRX-binding property of Txnip is unique [86]. Thus, the catalytic center of TRX seems to be important for the interaction. This interaction is important for cellular redox regulation, since the protein reducing activity of TRX is actually inhibited by the Txnip interaction [85,86]. In COS-7 and HEK293 cells transiently transfected with Txnip expression vector, a decrease in the insulin reducing activity of TRX and a diminishment of TRX expression was observed. In addition, treatment of HL-60 cells with 1α,25-dihydroxyvitamin D_3_ caused an increase in the Txnip expression and down-regulation of the expression and the reducing activity of TRX.

These results suggest that Txnip serves as a negative regulator of the biological function and expression of TRX by direct interaction, providing new insight into the redox-dependent signaling mechanism. We propose that this signal complex, composed of TRXs and Txnip as redox-dependent signal complexes, be known as “redoxisome”. We believe this signal complex could be a key regulatory mechanism for controlling various kinds of harmful stress (biostress) and preventing the progression or aggravation of stress diseases.

## Figures and Tables

**Table 1:** Thioredoxin as a marker for inflammatory disorders.

Disease	Sample	References
Acquired immunodeficiency syndrome (AIDS)	Plasma	[20,21]
Acute coronary syndrome	Serum	[22]
Acute myocardial infarction	Plasma	[23,24]
Acute pancreatitis	Serum	[25]
Acute respiratory distress syndrome (ARDS)	BALF/Plasma	[26]
Asthma	Serum	[27]
Atherosclerosis	Plasma	[28]
Burns	Serum	[29]
Cardiac surgery with cardiopulmonary bypass	Plasma	[30]
Chronic heart failure	Plasma	[31]
Diabetes mellitus	Plasma/Serum	[32,33]
Dilated cardiomyopathy	Serum	[22]
Hepatocellular carcinoma	Serum	[34,35]
Hepatitis C	Serum	[36]
Inflammatory bowel disease	Serum	[37]
Interstitial lung disease	Serum	[38]
Nonalcoholic steatohepatitis (NASH)	Serum	[39]
Non-small cell lung cancer	Serum	[40]
Obstructive sleep apnea (OSA)	Plasma	[41]
Oral squamous cell carcinoma	Saliva	[42]
Pancreatic ductal carcinoma	Plasma	[43]
Pulmonary sarcoidosis	BALF	[44]
Rheumatoid arthritis	Plasma/Serum/SF	[45,46,47]
Sjögren’s syndrome	Saliva	[48]
Subarachnoid hemorrhage	Serum	[49]
Trauma	Plasma	[50]
Unstable angina	Plasma	[51]

BALF: bronchoalveolar lavage fluid; SF: synovial fluid.
